# Substrate Selectivity of Coumarin Derivatives by Human CYP1 Enzymes: In Vitro Enzyme Kinetics and In Silico Modeling

**DOI:** 10.1021/acsomega.1c00123

**Published:** 2021-04-19

**Authors:** Risto O. Juvonen, Mira Ahinko, Elmeri M. Jokinen, Juhani Huuskonen, Hannu Raunio, Olli T. Pentikäinen

**Affiliations:** † School of Pharmacy, Faculty of Health Sciences, University of Eastern Finland, Box 1627, 70211 Kuopio, Finland; ‡ Department of Biological and Environmental Science & Nanoscience Center, University of Jyvaskyla, P.O. Box 35, FI-40014 Jyvaskyla, Finland; § Institute of Biomedicine, Faculty of Medicine, Integrative Physiology and Pharmacology, University of Turku, Kiinamyllynkatu 10, FI-20520 Turku, Finland; ∥ Department of Chemistry, University of Jyvaskyla, P.O. Box 35, FI-40014 Jyvaskyla, Finland

## Abstract

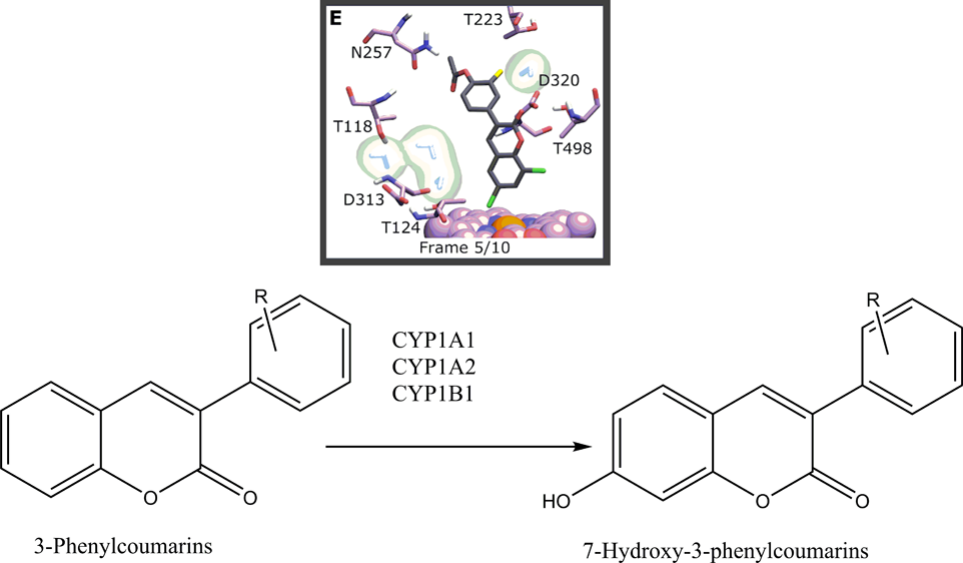

Of the three enzymes in the human cytochrome P450 family 1, CYP1A2 is an important enzyme mediating metabolism of xenobiotics including drugs in the liver, while CYP1A1 and CYP1B1 are expressed in extrahepatic tissues. Currently used CYP substrates, such as 7-ethoxycoumarin and 7-ethoxyresorufin, are oxidized by all individual CYP1 forms. The main aim of this study was to find profluorescent coumarin substrates that are more selective for the individual CYP1 forms. Eleven 3-phenylcoumarin derivatives were synthetized, their enzyme kinetic parameters were determined, and their interactions in the active sites of CYP1 enzymes were analyzed by docking and molecular dynamic simulations. All coumarin derivatives and 7-ethoxyresorufin and 7-pentoxyresorufin were oxidized by at least one CYP1 enzyme. 3-(3-Methoxyphenyl)-6-methoxycoumarin (**19**) was 7-O-demethylated by similar high efficiency [21–30 ML/(min·mol CYP)] by all CYP1 forms and displayed similar binding in the enzyme active sites. 3-(3-Fluoro-4-acetoxyphenyl)coumarin (**14**) was selectively 7-O-demethylated by CYP1A1, but with low efficiency [0.16 ML/(min mol)]. This was explained by better orientation and stronger H-bond interactions in the active site of CYP1A1 than that of CYP1A2 and CYP1B1. 3-(4-Acetoxyphenyl)-6-chlorocoumarin (**20**) was 7-O-demethylated most efficiently by CYP1B1 [53 ML/(min·mol CYP)], followed by CYP1A1 [16 ML/(min·mol CYP)] and CYP1A2 [0.6 ML/(min·mol CYP)]. Variations in stabilities of complexes between **20** and the individual CYP enzymes explained these differences. Compounds **14**, **19**, and **20** are candidates to replace traditional substrates in measuring activity of human CYP1 enzymes.

## 1 Introduction

Humans and other organisms are exposed to many foreign substances (xenobiotics). After absorption, xenobiotics are transformed by metabolizing enzymes to water-soluble and excretable metabolites. This biotransformation is the essential defense mechanism against lipophilic environmental substances.^1,2^ The most versatile enzymes catalyzing these reactions are members of the cytochrome P450 (CYP) superfamily. Especially, members of families CYP1, CYP2, and CYP3 catalyze functionalization reactions of xenobiotics and endogenous substances.^3,4^

The human CYP1 family comprises three forms: CYP1A1, CYP1A2, and CYP1B1, which differ particularly in structure and expression. However, they have not only many common but also some different substrate and inhibition properties due to their structural differences. The amino acid sequence of CYP1A2 is 72% identical to that of CYP1A1, while CYP1B1 has lower amino acid sequence identity with both CYP1A1 (38%) and CYP1A2 (37%). However, CYP1B1 is qualified as a CYP1 member on the grounds of similar substrate specificity and the common induction of CYP1s by the aryl hydrocarbon receptor (AHR). The AHR signaling pathway plays a role in several endogenous functions and processes.^5−7^

The X-ray crystal structures of CYP1A1,^8^ CYP1A2,^9^ and CYP1B1^10^ bound with α-naphthoflavone have been characterized. Although the sequence identity between CYP1A1 and CYP1A2 is greater than that between CYP1A1 and CYP1B1, the substrate-binding site of CYP1A1 is more similar to that of CYP1B1.^8^ CYP1A1 and CYP1B1 share similar binding site shapes, but four binding site amino acids are different between these enzymes, and the CYP1B1 binding site is smaller. Five amino acids differ in the binding sites of CYP1A2 and CYP1A1, and the side chains of these amino acids are generally larger in CYP1A2 than those in CYP1A1. However, the CYP1A2 binding site has an additional hydrophobic subcavity which is not seen in CYP1A1 and CYP1B1.

CYP1 enzymes play a critical role in the metabolism of both endogenous and exogenous substrates. A recent survey^11^ showed that CYP1A2 participates in the metabolism of 10% of all chemicals (drugs, physiological compounds, and general chemicals), whereas CYP1A1 and CYP1B1 are involved in the metabolism of 7 and 3% of all chemicals, respectively. Hepatic CYP1A2 is particularly important in metabolism of drugs, while extrahepatic CYP1A1 and CYP1B1 mediate metabolism of endogenous compounds. All three CYP1 family enzymes play a dominant role in metabolism (activation/inactivation) of chemical carcinogens.^12,13^

Catalytic activities of CYPs can be measured using profluorescent substrates, and these assays are simple, robust, and sensitive. The main challenge with profluorescent substrates is often their poor selectivity for the multiple CYP forms present in human and animal tissues.^14^ The classical substrate for CYP1 enzymes, 7-ethoxyresorufin, is profluorescent. However, its oxidation to fluorescent resorufin is almost equally well catalyzed by all three CYP1 enzymes.^13^ Oxidation of another in vitro probe substrate, 7-ethoxycoumarin, is catalyzed by multiple members of CYP1, CYP2, and CYP3 families.^15,16^

**Figure 1:**
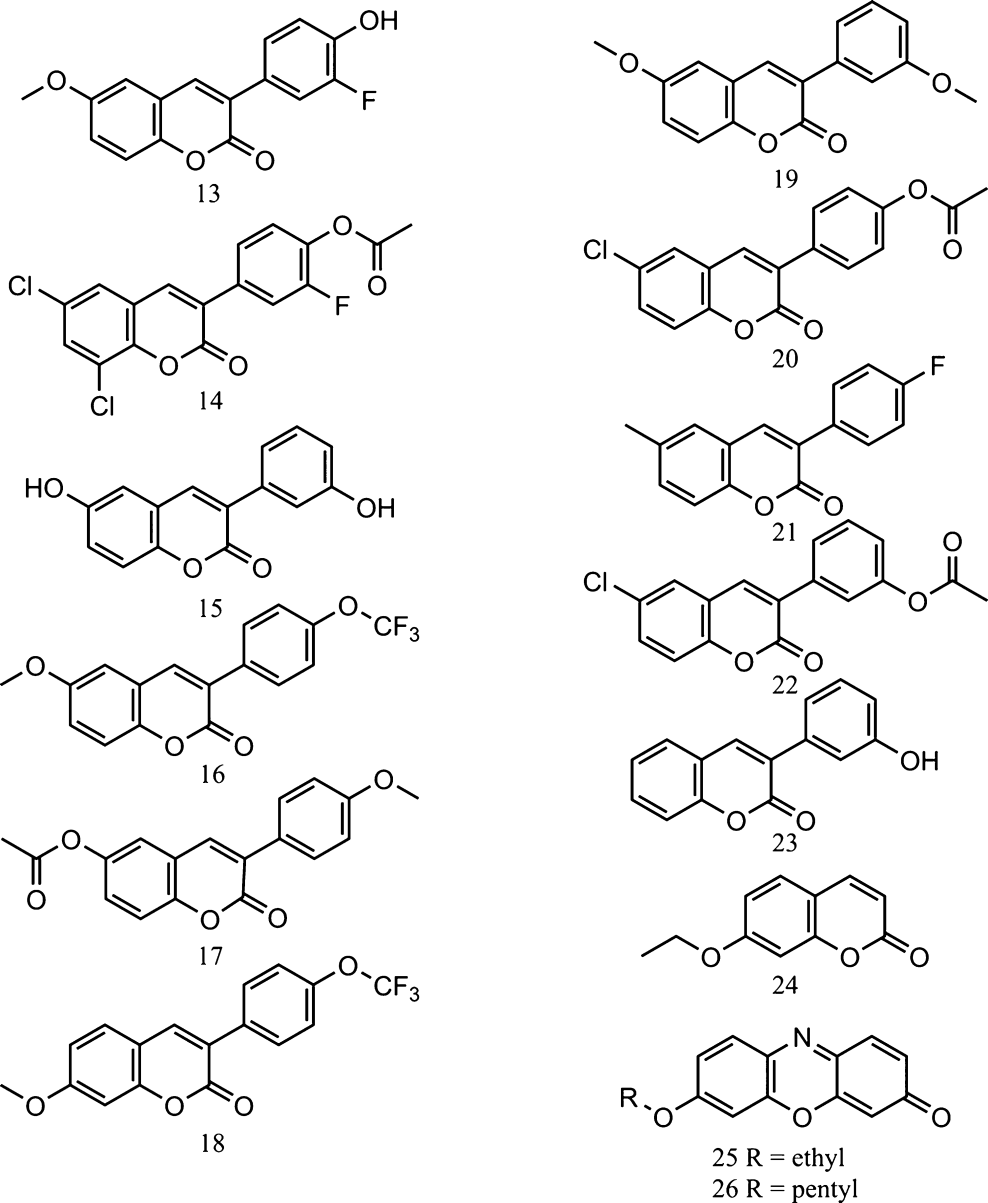
Structures of profluorescent substrates oxidized to fluorescent 7-hydroxycoumarin derivative metabolites. Compound **13**: 3-(3-fluoro-4-hydroxyphenyl)-6-methoxycoumarin, **14**: 3-(3-fluoro-4-acetoxyphenyl)coumarin, **15**: 3-(3-hydroxyphenyl)-6-hydroxycoumarin, **16**: 3-(4-trifluoromethoxyphenyl)-6-methoxycoumarin, **17**: 3-(4-methoxyphenyl)-6-acetoxycoumarin, **18**: 3-(4-trifluoromethoxyphenyl)-7-methoxycoumarin, **19**: 3-(3-methoxyphenyl)-6-methoxycoumarin, **20**: 3-(4-acetoxyphenyl)-6-chlorocoumarin, **21**: 3-(4-fluorophenyl)-6-methylcoumarin, **22**: 3-(3-acetoxyphenyl)-6-chlorocoumarin, **23**: 3-(3-hydroxyphenyl)coumarin, **24**: 7-ethoxycoumarin, **25**: 7-ethoxyresorufin, and **26**: 7-pentoxyresorufin.

Coumarin derivatives can be converted to fluorescent 7-hydroxycoumarin metabolites in an oxidation reaction typical to CYP enzymes.^17^ Recently, we described a general kinetic assay for profluorescent coumarin derivatives.^18^ In this assay, nonfluorescent coumarin derivatives are oxidized to their corresponding fluorescent 7-hydroxycoumarin derivatives. We noticed that several of these compounds were substrates of human CYP1A2 and often also of CYP1A1 or CYP1B1. The main aim of the present study was to find profluorescent coumarin substrates that are more selective for the individual CYP1 forms than the classical substrates 7-ethoxycoumarin and 7-ethoxyresorufin. To achieve this, we used the existing coumarin derivatives and synthesized new ones. Enzyme kinetic parameters of 12 coumarin derivatives and 7-ethoxycoumarin, 7-ethoxyresorufin, and 7-pentoxyresorufin were determined for CYP1A1, CYP1A2, or CYP1B1 (Figure 1). The interactions of these CYPs with the 3-phenylcoumarin substrates were evaluated with molecular dynamics (MD) simulations to reveal properties of their substrate selectivity.

## 2 Methods

### 2.1 Chemicals

Ethanol (≥99.5%, Etax Aa) was from Altia (Helsinki, Finland). Water was deionized by Milli-Q gradient A10. All chemicals were of the highest purity available from their commercial suppliers. Trichloroacetic acid, 7-ethoxycoumarin, Tris-HCl, MnCl_2_, MgCl_2_, reduced glutathione (GSH), isocitric acid, and isocitric acid dehydrogenase were purchased from Sigma-Aldrich (Steinheim, Germany), KCl was purchased from J.T. Baker, and NADPH and NADP^+^ were purchased from Roche Diagnostics (Mannheim, Germany). The NADPH regenerating system (200 mL) contained 178.5 mg of NADP^+^ (nicotinamide adenine dinucleotide phosphate), 645 mg of isocitric acid, 340 mg of KCl, 240 mg of MgCl_2_, 0.32 mg of MnCl_2_, and 15 U isocitric acid dehydrogenase.

### 2.2 Synthesis of Coumarin Derivatives

Eleven 3-phenylcoumarin derivatives were synthesized (Figure 1). Synthesis and experimental data for compounds **13–17**, **19**, and **21–23** have been published earlier.^19,20^ Compounds **15**(21) and **23**(22) have also been published by others prior to our studies. The readily fluorescent 7-hydroxy-3-(4-fluorophenyl)coumarin was used as a surrogate standard for quantification of metabolite formation.

Proton nuclear magnetic resonance (^1^H NMR) spectra were measured with a Bruker AVANCE (400 MHz) or a Bruker AVANCE III HD (300 MHz) spectrometer. The chemical shifts are expressed in parts per million (δ value) downfield from tetramethylsilane, using tetramethylsilane (δ = 0) and/or residual solvents such as chloroform (δ = 7.26) as an internal standard. Splitting patterns are indicated as follows: s, singlet; d, doublet; t, triplet; q, quartet; m, multiplet; and br; broad peak. Microwave heating was carried out with a CEM Discover microwave synthesizer. Elemental analyses were measured with Elementar Vario CHNOS.

**Scheme 1:**
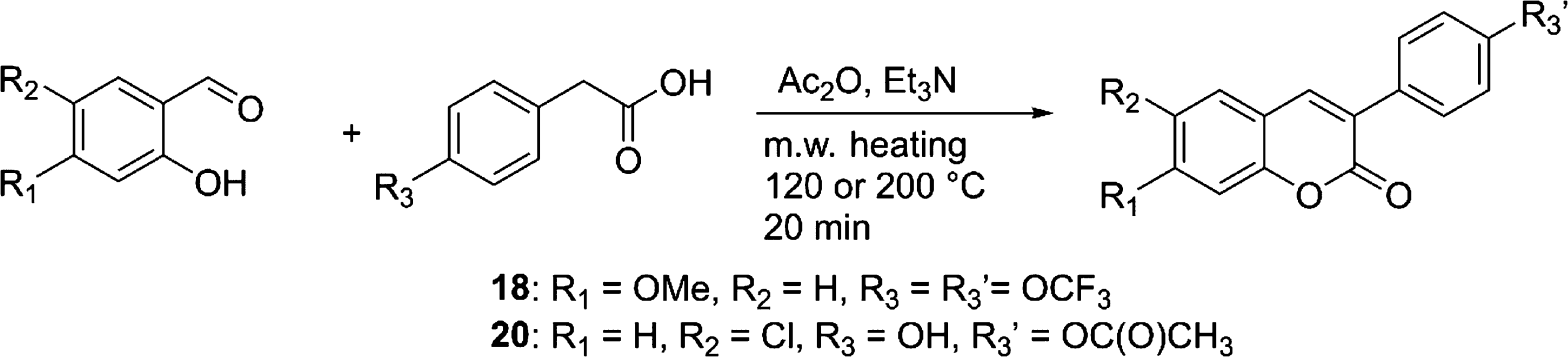


Synthesis of 7-methoxy-3-(4-(trifluoromethoxy)phenyl)-2*H*-chromen-2-one (**18**) (Scheme 1): 2-(4-(trifluoromethoxy)phenyl)acetic acid (173 mg, 0.79 mmol), 2-hydroxy-4-methoxybenzaldehyde (113 mg, 0.74 mmol), triethylamine (0.14 mL), and acetic anhydride (0.23 mL) were mixed in a microwave reactor tube (10 mL). The mixture was heated in a microwave reactor for 20 min at 200 °C. After cooling, the solid material was filtered and washed with cold ethanol. The raw product was recrystallized from ethanol–water, giving **18** as a pale solid (138 mg, 55%). ^1^H NMR (CDCl_3_, 400 MHz): δ 3.89 (s, 3H, OCH_3_), 6.86 (d, ^1^H, *J* = 2.4 Hz, H-8 (coumarin)), 6.88 (dd, ^1^H, *J* = 8.5, 2.4 Hz, H-6 (coumarin)), 7.26 (d, ^2^H, *J* = 8.7 Hz, H-3 (benzene)), 7.43 (d, ^1^H, *J* = 8.6 Hz, H-5 (coumarin)), 7.73 (d, ^2^H, *J* = 8.9 Hz, H-2 (benzene)), 7.76 (s, ^1^H, H-4 (coumarin)); ^13^C NMR (CDCl_3_, 100 MHz): δ 55.95, 100.61, 113.12, 113.25, 119.32, 120.94, 121.88, 123.50, 129.12, 130.04, 133.79, 140.50, 149.34, 155.57, 160.81, 163.07 (Figure S3); HRMS (ESI^+^): *m*/*z* [M + H]^+^ calcd for C_17_H_11_F_3_O_4_, 336.0609; found, 336.0612; Elemental analysis: calcd C % 60.72, H % 3.30, found C % 60.37, H % 3.29.

Synthesis of 4-(6-chloro-2-oxo-2*H*-chromen-3-yl)phenyl acetate (**20**) (Scheme 1): 2-(4-hydroxyphenyl)acetic acid (248 mg, 1.58 mmol), 2-chloro-2-hydroxybenzaldehyde (267 mg, 1.75 mmol), triethylamine (0.40 mL), and acetic anhydride (0.60 mL) were mixed in a microwave reactor tube (10 mL). The mixture was heated in a microwave reactor for 20 min at 120 °C. After cooling, the solid material was filtered and washed with cold ethanol and water. The raw product was recrystallized from ethanol–water, giving **20** as a pale solid (437 mg, 87%). ^1^H NMR (DMSO-*d*_6_, 300 MHz): δ 3.31 (s, ^3^H, COCH_3_), 7.23 (d, ^2^H, *J* = 9.0 Hz, H-3 (benzene)), 7.50 (d, ^1^H, *J* = 8.8, H-8 (coumarin)), 7.67 (d, ^1^H, *J* = 8.8, 2.6 Hz, H-7 (coumarin)), 7.76 (d, ^2^H, *J* = 9.0 Hz, H-2 (benzene)), 7.88 (d, ^1^H, *J* = 2.6 Hz, H-5 (coumarin)), 8.23 (s, ^1^H, H-4 (coumarin)); ^13^C NMR (CDCl_3_, 75 MHz): δ 21.28, 118.05, 120.76, 121.91, 127.25, 128.76, 129.90, 129.96, 131.54, 131.94, 138.54, 151.55, 152.02, 159.99, 169.38 (Figure S3); HRMS (ESI^+^): *m*/*z* [M + H]^+^ calcd for C_17_H_11_Cl_1_O_4_, 314.0346; found, 314.0344; Elemental analysis: calcd C % 64.88, H % 3.52, found C % 64.89, H % 3.62.

### 2.3 Biological Material

Baculovirus-insect cell-expressed human CYP1A1, CYP1A2, CYP1B1, CYP2A6, CYP2B6, CYP2C8, CYP2C9, CYP2C19, CYP2D6, CYP2E1, CYP3A4, CYP3A5, and CYP3A7 were purchased from BD Biosciences Discovery Labware (Woburn, MA, USA) and used according to the manufacturer’s instructions.

### 2.4 Oxidation Assays

The kinetic assays were carried out in 100 μL volume containing 100 mM Tris-HCl buffer pH 7.4, 0–40 μM coumarin derivative or 0–10 μM 7-ethoxyresorufin, 1–25 nM recombinant CYP or 0–0.1 g/L microsomal protein, and 20% NADPH regenerating system. Incubations took place at 37 °C in 96-multiwell plates; the fluorescence was measured with a Victor^2^ plate reader (PerkinElmer Life Sciences, Turku, Finland). The detailed conditions are described in the figures and tables. The reaction was started by adding NADPH, and fluorescence was measured at 2 min intervals for 40 min using excitation at 405 nm and emission at 460 nm for oxidation of coumarin derivatives^18^ and excitation at 570 nm and emission at 615 nm for ethoxyresorufin or pentoxyresorufin 7-O-dealkylations. Incubations without the substrate, enzyme, or NADPH were used as blank reactions. Resorufin was used as a standard, and 3-(4-fluorophenyl)-7-hydroxycoumarin was used as the surrogate standards to calculate the amount of product formed. The linear phase of the reactions was used for calculations.

Enzyme kinetic parameters were analyzed using the nonlinear Michaelis–Menten equation *v* = *V*_max_ × *S*/(*K*_m_ + *S*), in which *v* is the rate for 7-hydroxylation of a coumarin derivative, *S* is its concentration, and *V*_max_ value is the limiting rate of the reaction of 7-hydroxylation, which reached a half-maximal rate at a concentration of *K*_m_ for the substrate.

### 2.5 Molecular Modeling

Molecular docking and MD simulations were used to evaluate the structural basis of the interactions that facilitate 3-phenylcoumarin 7-hydroxylation catalysis by CYP forms 1A1, 1A2, and 1B1. Generally, a single docking pose, which was hypothesized to allow 7-hydroxylation, in each CYP1 form was selected for MD simulation for each compound. The binding energy, stability, and binding interactions of the compounds **13–23** (Figure 1) in their hypothesized 7-hydroxylation, facilitating binding modes in the CYP1 binding sites, were examined.

Compounds **13–26** were prepared for molecular docking. For **13–23**, partial charges were calculated for MD simulations. First, the compound 3D structures were prepared with the LigPrep module in the Schrödinger release 2020-1 (Schrödinger, LLC, New York, NY, 2020). Protonation was performed using target pH 7.4, ionization and tautomerization were carried out using Epik^23^ with the metal-binding option, a maximum of eight tautomers were generated, and the OPLS_2005 force field was used for partial charges and geometry optimization. The 3D structure preparation resulted in one output structure per compound. Second, the 3D structures of the compounds were geometry-optimized quantum mechanically with Gaussian 16^24^ at the HF/6-31+G(d) level using the polarizable continuum model. The final atom-centered partial charges for the compounds were derived from the electrostatic potentials and applied using the RESP method.^25^

The CYP enzyme 3D structures were prepared for molecular docking. The X-ray crystal structures were retrieved from the RCSB Protein Data Bank.^26^ The available α-naphthoflavone-bound structures were used for CYP1A1 (PDB code 4I8V),^8^ CYP1A2 (PDB code 2HI4),^9^ and CYP1B1 (PDB code 3PM0).^10^ A short gap in a loop of amino acid residues 308–311 of CYP1B1 was filled using homology modeling. The full CYP1B1 sequence was retrieved from the UniProt database (sequence code Q16678-1^27^). The full sequence was aligned to the CYP1B1 crystal structure sequence using Malign^28^ in the Bodil modeling environment^29^ with the structure-based matrix STRMAT110^28^ and gap formation penalty 40. The homology model was built with Nest.^30^ Finally, the water molecules and the bound ligand were removed from each CYP1 3D structure, and protons were added using Reduce 3.24.^31^

Compounds **13–26** were docked to the prepared CYP1 3D structures using Plants 1.2^32^ and the ChemPLP^33^ scoring function. The binding site center was defined based on the bound ligand, and the binding site radius was set to 10.0 Å. Eight docking poses per compound were output with cluster root mean square deviation (rmsd) set to 3.0 Å. For each compound, one docking pose was selected for consequent MD simulation. The selection was based on the hypothesized binding mode from our previous study.^18^ It was hypothesized that the 3-phenylcoumarin binding mode most suitable for 7-hydroxylation would have the 2-carbonyl toward Ser122 (CYP1A1), Thr124 (CYP1A2), or Ala133 (CYP1B1), where the 2-carbonyl could form a hydrogen bond (H-bond) with CYP1A1 and CYP1A2. In the docking pose selection, the position 7 or the methyl carbon in the 7-methoxy group of **18** was required to be within 6 Å of the CYP heme iron. The 2-carbonyl was required to orient toward Ser122, Thr124, or Ala133 in CYP1A1, CYP1A2, and CYP1B1, respectively. An exception was made on **14**, which has protective chlorine substituents at positions 6 and 8. Thus, it was hypothesized that in the abovementioned binding mode, the 8-chlorine would shield position 7 from oxidation. An alternative binding pose was selected for **14**, where the 2-carbonyl is toward the conserved Asp320/333 (CYP1A/1B1) and Thr497/498/510 (CYP1A1/1A2/1B1).

The ligand–protein complexes were prepared for the MD simulations from the selected docking poses. The original X-ray crystal structure water molecules farther than 4.0 Å from the heavy atoms of each docked compound were added back to the ligand–protein complex structure. LEaP in Amber 18^34^ package was used to protonate, solvate, apply force fields, and neutralize the ligand–protein complexes. Where applicable, the system was neutralized with Na^+^ or Cl^–^ ions. For each complex, a rectangular box was filled with transferable intermolecular potential three-point (TIP3P) water molecules extending 13.0 Å around the solute. The ff14SB force field^35^ was used for the protein, and all-atom parameters suitable for a six-coordinate iron were used for the heme group.^36^ Ligand parameters were derived from the GAFF force field,^37^ and the partial charges were derived with the RESP method as described above.

NAMD 2.13^38^ was used for MD simulations. A three-step equilibration process was employed: (1) 15,000 steps of energy minimization with the protein backbone Cα atoms constrained (5 kcal/mol), (2) 15,000 steps of energy minimization with no constraints, and (3) 1,200,000 steps (2.4 ns) of MD simulation with the Cα atoms constrained (5 kcal/mol). Finally, 12,000,000 steps (24 ns) of production MD simulation with no constraints were run. The detailed settings were employed as described earlier.^39^

Numerical analysis was performed on 200 frames, and visual analysis was performed on 10 frames of each MD simulation. The visual analysis was performed using Bodil.^29^ Cpptraj^40^ in Amber 18 was used to calculate rmsd values, atomic pairwise distances, the count of ligand–water H-bonds, and the amount of water molecules within 3.4 Å of the ligand. The rmsd of the ligand and the heme (rmsd_LH_) were calculated together in order to (1) consider the orientation of the ligand in relation to the CYP binding site and still (2) diminish the effect of the protein macromovement to the superpositioning of the ligand. Atomic pairwise distances were calculated between the heme iron and **13–23** position 7 or the carbon in the 7-methoxy group of **18**. Binding energies were calculated using the Nwat-MMGBSA method^41−43^ with *N* = 10 and *N* = 20, where *N* is the count of the closest water molecules to the ligand. The binding energy calculation was performed using the MMPBSA.py^44^ in the Amber 18 package with igb5.^45^

## 3 Results

### 3.1 CYP1 Oxidation of the Coumarin Derivatives

The coumarin compounds studied here are an extension of our previous study, in which 3-phenylcoumarin derivatives were demonstrated to be convenient profluorescent probe substrates for several human CYP forms.^18^ One of the conclusions of the study was that 3-phenylcoumarin is an optimal scaffold to design profluorescent substrates for the human CYP1A1, CYP1A2, and CYP1B1 enzymes. In the present study, CYP oxidation selectivity and Michaelis–Menten parameters were determined for the 11 new 3-phenylcoumarin compounds **13–23** and the traditional substrates 7-ethoxycoumarin (**24**), 7-ethoxyresorufin (**25**), and 7-pentoxyresorufin (**26**) (Figure 1), and their interactions with the CYP1 enzymes were studied by docking and MD simulations. The goals were to find more selective profluorescent substrates for the individual CYP1 forms and to identify ligand–enzyme interactions that contribute to form selectivity.

**Figure 2:**
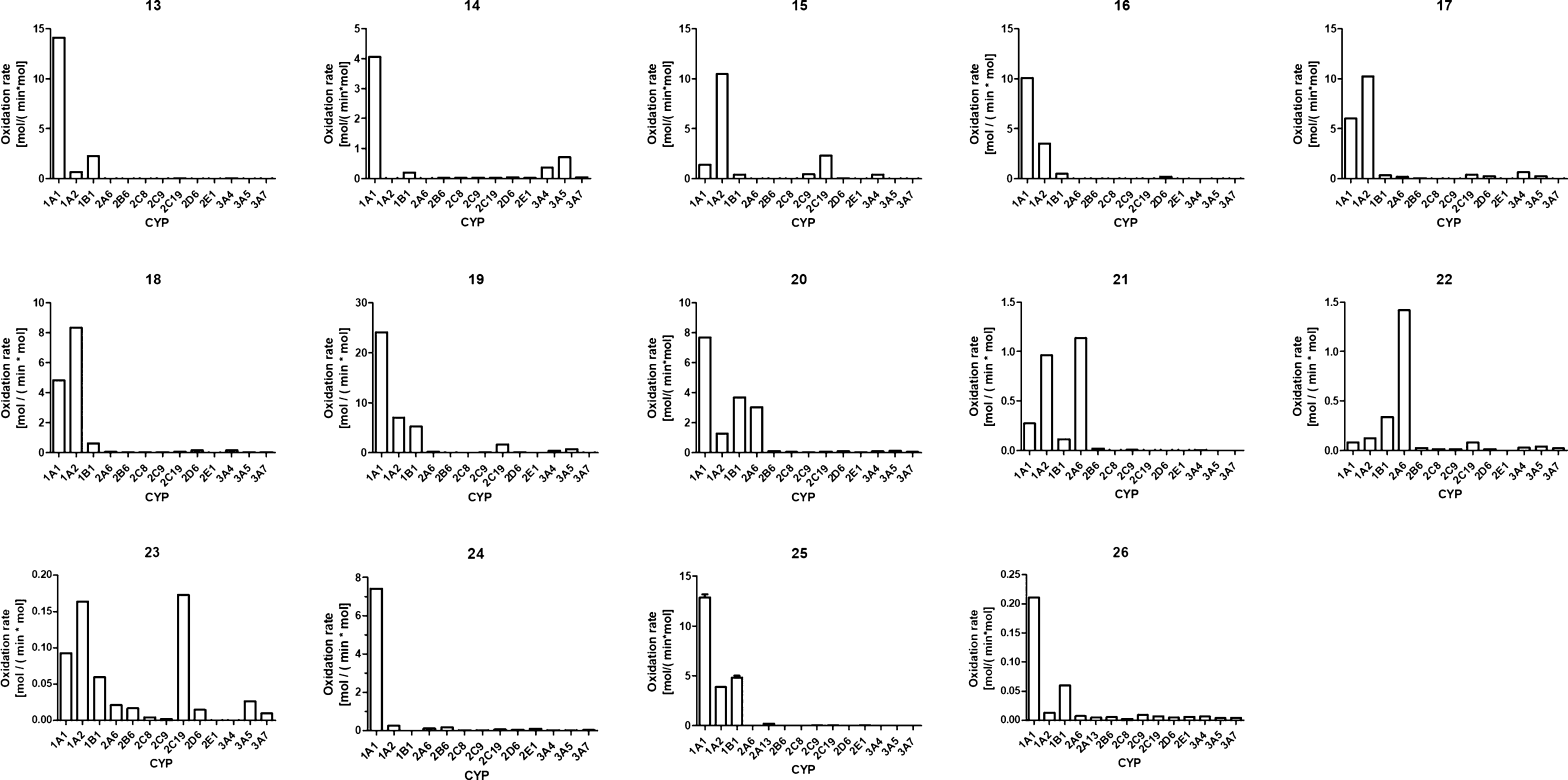
Oxidation of the coumarin derivatives and 7-ethoxy- and 7-pentoxyresorufin by 13 human CYP forms. The formation of fluorescent metabolites was determined in incubations containing a 25 nM CYP, a 20% NADPH regenerating system, and a 10 μM coumarin derivative or 1 μM 7-ethoxy/7-pentoxyresorufin in 100 mM Tris-HCl pH 7.4 in the kinetic experimental setup. The rates’ unit is mol product/(min·mol CYP) and does not represent *V*_max_ values. Note the variation in *Y*-axis scales between subpanels.

To find out which CYP forms oxidize the 11 novel coumarin derivatives, we determined their oxidation rates to the corresponding 7-hydroxycoumarin metabolites by 13 recombinant human CYP enzymes at a fixed 10 μM substrate concentration (Figure 2). All except **21**, **22**, and **23** were oxidized faster by one or several CYP1 forms than by the other CYP forms. Compounds **13**, **14**, **16**, **19**, and **20** were oxidized faster by CYP1A1 than CYP1A2 or CYP1B1; **15**, **17**, **18**, **21**, and **23** were oxidized faster by CYP1A2 than CYP1A1 or CYP1B1; and **22** was oxidized faster by CYP1B1 than by CYP1A1 or CYP1A2. Oxidation rates of the classical CYP substrates 7-ethoxycoumarin, 7-ethoxyresorufin, and 7-pentoxyresorufin were also determined. All these substrates were oxidized faster by CYP1A1 than the other CYP1 enzymes. Oxidation of 7-ethoxycoumarin and 7-ethoxyresorufin was 35 and 60 times faster, respectively, than oxidation of 7-pentoxyresorufin. 7-Ethoxyresorufin was oxidized by CYP1A2 and CYP1B1 at 30–40% of the rate of CYP1A1.

Due to the use of a surrogate standard to calculate the 7-hydroxylation rates, it was not possible to directly compare the rates between different coumarin derivatives, but comparison among CYPs was reliable. The rate varied from 0.20 to 24 mol/(min·mol CYP). Fluorescence change and the rate of the oxidation were the lowest for **23** and **26**, almost equally low for **21** and **22**, whereas the rate was high for the rest of the compounds. It can be concluded that most of these coumarin derivatives and 7-ethoxy- and 7-pentoxyresorufins are, to some extent, selective substrates for CYP1 enzymes.

Enzyme kinetics of 7-hydroxylation or 7-O-dealkylation were determined for all coumarin derivatives and for 7-ethoxyresorufin and 7-pentoxyresorufin. Because 7-ethoxyresorufin, 7-pentoxyresorufin, and 7-ethoxycoumarin are established substrates of CYP1 enzymes, their *K*_m_ and *V*_max_ values were determined for comparison. The *K*_m_ and *V*_max_ values and the calculated intrinsic clearances (*V*_max_/*K*_m_) are summarized in Table 1 and Figure S1. *K*_m_ values varied between 0.054 (**25**) and 15.6 (**14**) μM for CYP1A1, 0.27 (**19**)–47 (**23**) μM for CYP1A2, and 0.095 (**20**)–120 (**24**) μM for CYP1B1. *V*_max_ of the oxidation reaction varied from 1.35 (**26**) to 32 (**13**) mol/(min·mol CYP) for CYP1A1, 0.25 (**26**)–29.8 (**15**) mol/(min·mol CYP) for CYP1A2, and 0.29 (**15**)–9.3 (**19**) mol/(min·mol CYP) for CYP1B1. Substrates having low *K*_m_ had high intrinsic clearance, indicating high efficiency of oxidation (reciprocal value of *K*_m_ vs intrinsic clearance *r*^2^ = 0.839). Intrinsic clearance could not be determined for **21**, **22**, and **23** because of their low oxidation rates.

**Table 1:** Michaelis–Menten Constants of CYP1 Enzyme-Catalyzed Oxidation Reactions^a^

	CYP1A1			CYP1A2			CYP1B1		
compound	*K*_m_	*V*_max_	*V*_max_/*K*_m_	*K*_m_	*V*_max_	*V*_max_/*K*_m_	*K*_m_	*V*_max_	*V*_max_/*K*_m_
**13**	10.7 (2.6–18.7)^b^	32 (18–46)	3.0	20.3 (0–53)	6.0 (0–12.8)	0.29	7.8 (3.0–2.6)	6.9 (4.7–9.2)	0.89
**14**	15.6 (10.5–20.6)	2.5 (2.1–2.9)	0.16		NA^c^			LA^d^	
**15**	2.11 (0.17–4.1)	4.0 (3.0–5.0)	1.9	2.4 (1.8–3.1)	29.8 (27.5–32.2)	12.3	0.53 (0–1.1)	0.29 (0.25–0.34)	0.55
**16**	4.8 (2.6–6.9)	12.6 (10.6–14.6)	2.6	0.96 (0.61–1.32)	4.4 (4.1–4.8)	4.6		LA	
**17**	1.04 (0.65–1.42)	7.5 (7–8.1)	7.2	2.2 (1.6–2.8)	8.7 (8–9.4)	4.0		LA	
**18**	9.7 (4.8–14.6)	4.2 (3.3–5.1)	0.43	7.9 (5.1–10.6)	9.1 (7.8–10.5)	1.2		LA	
**19**	0.96 (0.4–1.52)	24.2 (19.4–29)	25.2	0.27 (0.19–0.36)	8.2 (7.6–8.9)	30.4	0.43 (0.24–0.61)	9.3 (8.2–10.4)	21.6
**20**	0.41 (0.26–0.55)	6.5 (5.9–7.1)	16	2 (1.2–2.7)	1.2 (1.1–1.4)	0.6	0.095 (0.04–0.15)	5.1 (4.7–5.5)	53
**21**		LA		7.6 (0.59–14.6)	1 (0.63–1.38)	0.13		LA	
**22**		LA			LA		1.1 (0.5–1.7)	1.1 (0.96–1.2)	1.0
**23**		LA		47 (8–86)	0.4 (0.19–0.6)	0.0085		LA	
**24**	11.9 (0–25.1)	26.2 (14.5–38)	2.21	7.7 (1.4–14)	2.0 (1.4–2.6)	0.26	120 (0–282)	1.75 (0–3.54)	0.015
**25**	0.054 (0.035–0.075)	10.3 (9.3–11.4)	191	0.56 (0.44–0.68)	3.2 (2.9–3.6)	5.8	0.096 (0.072–0.119)	3.3 (3.0–3.6)	34
**26**	0.74 (0.54–0.94)	1.35 (1.19–1.50)	1.82	0.93 (070–1.17)	0.25 (0.22–0.28)	0.27	2.59 (1.65–3.53)	0.37 (0.28–0.46)	0.14

a Units are μM for *K*_m_, mol/(min·mol CYP) for *V*_max_, and ML/(min·mol CYP) for *V*_max_/*K*_m_.

b 95% confidence interval.

c NA, no activity was observed.

d LA, low screening activity at the 20 μM substrate and 25 nM CYP concentration.

The coumarin derivatives **13**, **14**, and 7-ethoxycoumarin, 7-ethoxyresorufin, and 7-pentoxyresorufin were efficient substrates for CYP1A1 compared with CYP1A2 and CYP1B1. Compound **15** was an efficient substrate for CYP1A2 compared with CYP1A1 and CYP1B1, and **20** was an efficient substrate for CYP1B1 compared with CYP1A1 and 1A2. Compound **19** was oxidized with equal efficiency by all CYP1 enzymes. Compounds **16** and **18** were oxidized with equal efficiency by CYP1A1 and CYP1A2 and weakly by CYP1B1 (Table 1).

### 3.2 Docking and MD Simulations

Compounds **13–26** were first docked to CYP1A1 (PDB code 4I8V), CYP1A2 (PDB code 2HI4), and CYP1B1 (PDB code 3PM0) to determine their orientation in the active sites. In the selected docking pose, all 3-phenylcoumarins were oriented so that the coumarin position 7 or the methyl carbon in the 7-methoxy group of **18** was within 6.0 Å of the heme iron. The 2-carbonyl of **13** and **15–23** was located toward CYP1A1 Ser122 or Asp313, toward CYP1A2 Thr124 or Asp313 or toward CYP1B1 Ala133 or Asp326. The 2-carbonyl of **14** was oriented toward CYP1A1 Asp320 and Thr497, CYP1A2 Asp320 and Thr498, or CYP1B1 Asp333 and Thr510. In compounds **24–26**, docking identified poses where 2-carbonyl was orientated toward CYP1A1 Ser116, CYP1A2 Thr118, or CYP1B1 Ser127. Generally, the aliphatic hydrocarbon chain at coumarin position 7 elevates the core structure in comparison to the 3-phenyl compounds whose coumarin core resides closer to the heme (Figure S2). Recently, we made a similar observation in the predicted binding mode of 3-phenyl-coumarins and 7-ethoxycoumarin with CYP2A13.^46^ Another major difference between **13–23** and the traditional substrates **24–25** is that the 3-phenyl-group increases the size of the compounds, resulting in selectivity toward different CYP forms using amino acid similarities and differences between the forms. For example, in addition to interacting with residues closer to heme, these new substrates reach the area containing similar residues in the CYP1 family (CYP1A1: Asn255; CYP1A2: Asn257; and CYP1B1: Asn265) but are different in CYP2 (hydrophobic residue) and CYP3 families (open space). To develop form selectivity of substrates within the CYP1 family, introduction of slight variations in critical positions would be sufficient. Substrates that can use sequence differences next to heme, jointly with more distant positions (such as CYP1A1: Asn222; CYP1A2: Thr223; and CYP1B1: Asn228), may yield smaller nuances to CYP1 form selectivity.

The selected docking poses were subjected to MD simulations. In MD simulations, the Nwat-MMGBSA-calculated binding energies varied from −29.4 to −49.9 kcal/mol (*N* = 10), which was almost equal to *N* = 20 calculations (Table S1). The presence of water molecules had a minimal effect on the binding energy differences between *N* = 10 and *N* = 20 in general because so few water molecules were within 3.4 Å of the substrates. However, in most cases, substrate-water H-bonds took place and affected binding (Table 2). In the MD simulations, the general orientation of the compounds did not change markedly from the original starting positions (Table S2). Position 7 of **13–23** remained mainly within 6.0 Å of the CYP heme iron (Table 3). A longer distance of the 2-carbonyl from the Ser122 (CYP1A1) and Thr124 (CYP1A2) hydroxyl oxygen atom or the Ala133 methyl carbon atom (CYP1B1) suggested upright orientations of the compounds in relation to heme (Table 3).

**Table 2:** Count and Standard Deviations of 3-Phenylcoumarin Interactions with Water Molecules in the CYP1 Binding Sites

	waters within 3.4 Å of the ligand			ligand–water H-bonds		
compound	CYP1A1	CYP1A2	CYP1B1	CYP1A1	CYP1A2	CYP1B1
**13**	4.5 ± 1.9	0.9 ± 0.3	4.4 ± 1.8	0.9 ± 0.6	0.7 ± 0.4	1.8 ± 0.8
**14**	3.5 ± 1.0	1.6 ± 0.9	4.0 ± 1.0	1.3 ± 0.7	0.1 ± 0.2	0.9 ± 0.6
**15**	7.4 ± 2.2	2.7 ± 1.4	4.3 ± 1.4	2.2 ± 1.2	1.1 ± 0.9	1.6 ± 0.7
**16**	5.2 ± 1.8	1.8 ± 0.7	4.3 ± 1.8	0.9 ± 0.6	0.7 ± 0.5	0.7 ± 0.5
**17**	5.2 ± 2.0	2.7 ± 0.7	3.9 ± 1.1	1.4 ± 0.8	1.4 ± 0.7	1.4 ± 0.8
**18**	3.7 ± 1.1	3.2 ± 2.0	3.3 ± 1.2	0.0 ± 0.1	0.7 ± 0.5	0.0 ± 0.0
**19**	7.0 ± 1.8	1.9 ± 0.6	4.1 ± 1.5	0.9 ± 0.5	0.8 ± 0.4	1.1 ± 0.7
**20**	4.2 ± 1.3	4.0 ± 1.5	4.2 ± 0.8	0.7 ± 0.6	0.8 ± 0.6	1.4 ± 0.6
**21**	4.8 ± 1.3	1.6 ± 0.6	4.3 ± 1.2	1.1 ± 0.6	0.8 ± 0.4	0.9 ± 0.4
**22**	4.5 ± 1.5	2.0 ± 1.4	2.7 ± 0.8	1.0 ± 0.6	0.8 ± 0.6	0.5 ± 0.5
**23**	3.2 ± 1.0	2.2 ± 0.8	5.3 ± 1.3	0.0 ± 0.2	0.9 ± 0.6	2.0 ± 0.8
**average**	4.8	2.2	4.1	1.0	0.8	1.1

**Table 3:** Distance (Å) of 3-Phenylcoumarin Positions 2 and 7 to Selected CYP1 Residues with Standard Deviations

	position 7 to heme iron			position 2 carbonyl to S122 (CYP1A1) or T124 (CYP1A2) hydroxyl oxygen or A133 methyl carbon (CYP1B1)		
compound	CYP1A1	CYP1A2	CYP1B1	CYP1A1	CYP1A2	CYP1B1
**13**	4.5 ± 0.4	5.0 ± 0.6	4.9 ± 0.8	3.2 ± 0.8	5.4 ± 0.8	6.0 ± 1.2
**14**	4.1 ± 0.3	5.1 ± 0.3	4.6 ± 0.5	8.0 ± 0.5	9.8 ± 0.4	9.1 ± 0.8
**15**	5.5 ± 0.9	4.3 ± 0.5	5.2 ± 0.4	6.9 ± 1.1	5.3 ± 0.7	4.1 ± 0.5
**16**	4.5 ± 0.7	5.4 ± 0.4	4.5 ± 0.4	4.9 ± 1.5	6.3 ± 0.5	5.2 ± 1.0
**17**	4.7 ± 0.6	5.3 ± 0.5	5.1 ± 0.4	5.2 ± 1.1	6.3 ± 0.6	6.6 ± 0.5
**18**	4.1 ± 0.6	4.1 ± 0.5	4.4 ± 0.5	2.8 ± 0.3	6.5 ± 0.6	3.6 ± 0.3
**19**	5.0 ± 0.5	4.7 ± 0.3	6.8 ± 0.7	5.8 ± 0.8	5.1 ± 0.5	8.0 ± 0.8
**20**	3.9 ± 0.4	4.6 ± 0.5	4.4 ± 0.4	3.2 ± 0.7	5.1 ± 1.0	5.4 ± 0.5
**21**	4.4 ± 0.5	4.7 ± 0.5	4.8 ± 0.5	5.9 ± 0.7	5.8 ± 0.7	6.0 ± 0.7
**22**	4.3 ± 0.5	6.1 ± 1.2	4.4 ± 0.4	3.5 ± 0.8	6.7 ± 1.4	3.4 ± 0.3
**23**	4.5 ± 0.5	4.0 ± 0.4	4.7 ± 0.5	2.8 ± 0.4	5.0 ± 0.6	4.8 ± 0.6
**average**	4.5	4.8	4.9	4.8	6.1	5.6

**Figure 3:**
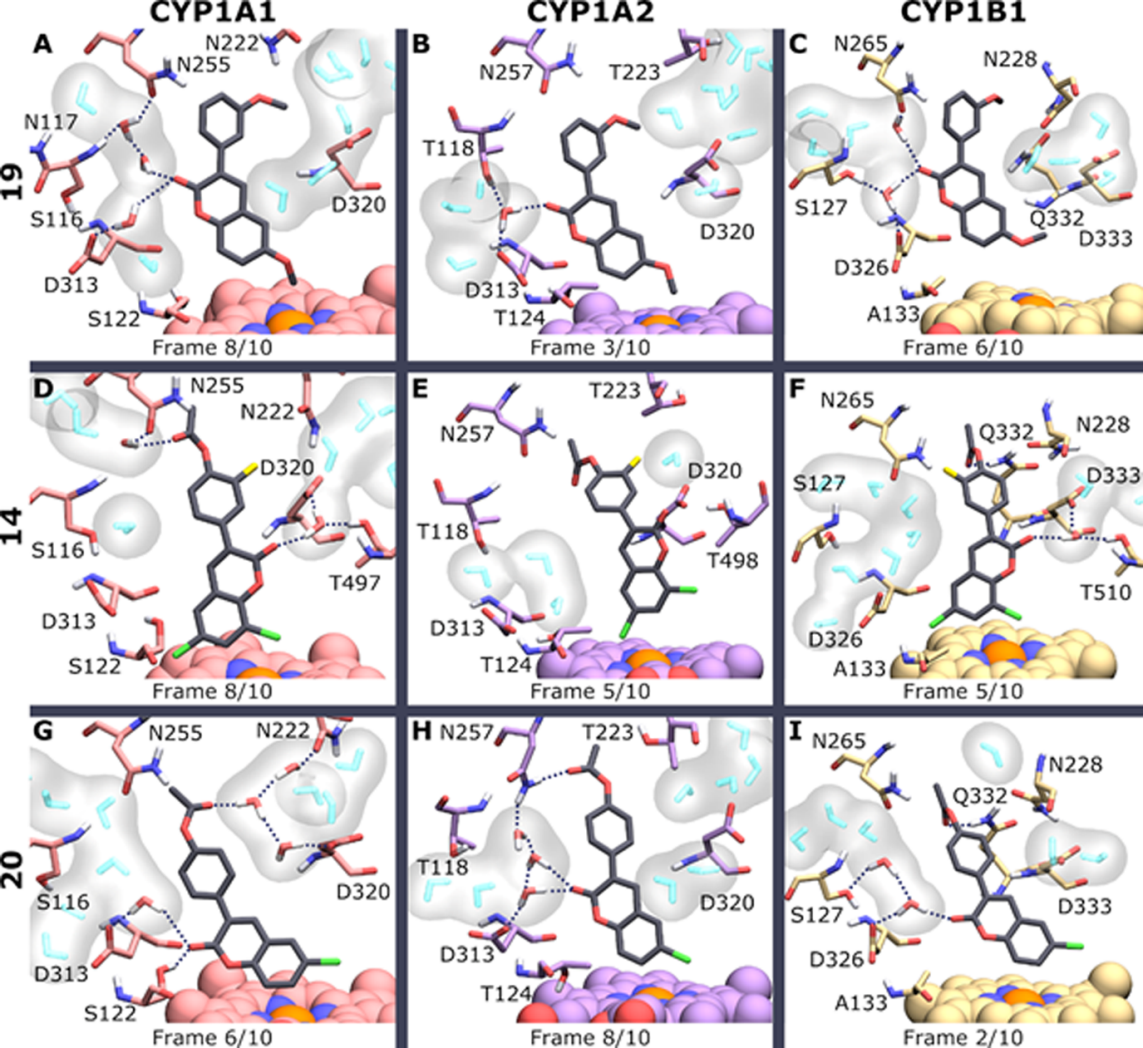
Representative snapshots of MD simulations with three coumarin derivatives and human CYP1s. Compounds 19 (A–C), 14 (D–F), and 20 (G–I) (gray stick models) in complex with CYP (stick models for amino acids and van der Waals models for the heme) form 1A1 [pink; (A,D,G)], 1A2 [violet; (B,E,H)], and 1B1 [light yellow; (C,F,I)]. The snapshots were selected from 10 visually inspected frames of each corresponding 24 ns MD simulation. Red: oxygen; blue: nitrogen; white: hydrogen; orange: iron; yellow: fluorine; and green: chlorine. Only those water molecules (cyan or atomically colored stick models with a gray surface) that are both among the 20 closest ones to the ligand and connected to the CYP1 binding site are shown. H-bonds (dotted lines) are visualized for direct ligand–protein bonds and interactions mediated by one or two water molecules.

The MD simulations and interactions of **14**, **19**, and **20** in complex with CYP1A1, CYP1A2, and CYP1B1 were investigated in detail to obtain more information about the 7-hydroxylation selectivity. These compounds were selected because **19** was oxidized with similar efficiency by all CYP1 enzymes, **14** was oxidized selectively by CYP1A1, and **20** was oxidized selectively by CYP1B1 (Table 1). The orientation of **19** in the binding pocket of CYP1s was similar so that **19** was in a vertical position toward heme and the 7-position was toward the iron (Figure 3A–C), having the lowest distance in CYP1A2 (Table 3). H-bonds existed between the carbonyl oxygen and Asp313, Ser116, and Asn255 of CYP1A1, Asp313 and Thr118 of CYP1A2, and Ser127, Asn265, and Asp326 of CYP1B1, in which water molecules played an important role (Figure 3A–C). The interactions of **19** in the CYP1B1 binding site resembled those in CYP1A1 and differed from the ones in CYP1A2. The **19**-enzyme complexes were about equally stable between all three CYP1 forms. However, the binding mode was the most stable in CYP1A2 with a perfectly stabilized H-bond via water, while some waters slightly disrupted the binding mode in CYP1A1 and CYP1B1. Similar interaction and binding poses in MD simulations of **19** with CYP1 forms (Figure 3A–C) are in line with similarly low *K*_m_ values and high 7-hydroxylation catalytic efficiency of all these enzymes.

Compound **14** is larger than **19**, since it has 6,8-dichlorine, 3′-fluorine, and 4′-acetoxy substituents instead of only 6,3′-dimethoxy in **19**. It was moderately efficiently oxidized by CYP1A1 and very weakly by CYP1B1 and not at all by CYP1A2 (Figure 2, Table 1). The orientation of **14** in the binding pocket of CYP1A2 differed from the orientation in CYP1A1 and CYP1B1 (Figure 3D–F), and complex formation was less stable in CYP1A2 than in CYP1A1 and CYP1B1. In the CYP1A2 binding pocket, 6-chlorine was oriented toward heme iron, thus suggesting prevention of oxidation of the 7-position (Figure 3E). In contrast, in CYP1A1 and CYP1B1, the 7-position was oriented toward heme iron (Figure 3D,F, respectively), which could indicate easier oxidation of the 7-position. However, chlorines at 6- and 8-positions potentially shield the 7-position from efficient oxidation. Carbonyl oxygen formed a H-bond via the water molecule with Asp320 and Thr497 of CYP1A1 and Asp333 and Thr510 of CYP1B1. H-bonds were formed from 4′-acetoxy to Asn255 and Asp313 of CYP1A1 via water and to Gln332 of CYP1B1. The orientation of **14** and its interactions between CYP1s are in line with the 7-hydroxylation differences between these enzymes.

Compound **20** was oxidized to 7-hydroxyl metabolites by all human CYP1s but with different efficiencies. CYP1B1 was the most efficient with the lowest *K*_m_ followed by CYP1A1 and CYP1A2. The orientation of **20** in the binding pocket of all CYP1s was similar so that **20** was in a vertical position toward the heme and the 7-position was toward the iron (Figure 3G–I), having the lowest distance in CYP1A1 (Table 3). However, in CYP1A2, chlorine at 6-position is adjacent to the 7-position toward heme iron, while in CYP1A1 and CYP1B1, 7- and 8-positions are toward heme iron. This difference is likely due to Thr124 in CYP1A2, which blocks binding into a similar orientation to that with CYP1A1 and CYP1B1. Accordingly, in CYP1A2, chlorine in **20** might shield 7-position more efficiently from oxidation than in CYP1A1 and CYP1B1. The order of interaction stability between **20** and CYPs was CYP1B1, CYP1A1, and CYP1A2. Asp326 and Gln332 of CYP1B1 formed H-bonds with carbonyl oxygen and 4′-acetoxy, respectively. The H-bonds of **20** in CYP1A1 and CYP1A2 were disturbed by water molecules, which decreased the stability of **20** in their binding pockets. The decrease in stability was greater in CYP1A2 than in CYP1A1. Binding stability of **20** better explained differences in its *K*_m_ and the 7-hydroxylation efficiencies than its orientation in the enzymes. The supplement contains more detailed description of interactions between coumarin derivatives and the CYP1 enzymes.

## 4 Discussion

The most common profluorescent probe substrates of CYP1 enzymes have been 7-ethoxycoumarin and 7-ethoxyresorufin. 7-Ethoxycoumarin is oxidized by both human CYP1A1 and CYP1A2, whereas 7-ethoxyresorufin is oxidized by all CYP1 forms (CYP1A1, CYP1A2, and CYP1B1). We report here several new profluorescent coumarin substrates of human CYP1A1, CYP1A2, and CYP1B1. Compound **19** was 7-O-demethylated by similar high efficiency by all CYP1 forms, **14** was selectively 7-hydroxylated, but with low efficiency by CYP1A1, and **20** was 7-hydroxylated most efficiently by CYP1B1. The 2-carbonyl substituent is important for their interaction and metabolically optimum orientation in the binding site of the CYP1s. In CYP1A1, the residue Ser122 formed important H-bonding, which did not take place in CYP1A2 and CYP1B1. Another important H-bond was mediated by water from the 3-phenylcoumarin 2-carbonyl to the CYP1-conserved Asp313 (CYP1A1 and CYP1A2) or Asp326 (CYP1B1).

Several modeling approaches have been used to evaluate ligand–enzyme interactions of human CYP1A1 and CYP1A2.^47^ Both enzymes bind mainly planar ligands, but CYP1A1 has been shown to prefer linear molecules, while CYP1A2 prefers triangular molecules. In addition to optimal H-bonding groups, π–π stacking interactions between ligands and amino acid residues at the active sites in these enzymes are important. In general, multiple binding modes can exist for a ligand within a specific CYP form, which can partly explain different metabolic and inhibitory activities of different ligands and CYP enzymes. For example, Liu et al.^48^ observed that the preferred docking-produced binding mode of 3-phenyl-substituted 7-ethynylcoumarin derivatives was different based on whether the compounds were competitive inhibitors or mechanism-based inactivators for CYP1A2. Similar relation has been observed in MD simulations of 7-methylcoumarin with CYP2A6 and CYP2A5,^49^ and *N*-(3,5-dichlorophenyl)cyclopropane-carboxamide and α-naphthoflavone with CYP1A1 and CYP1A2.^50^ Similarly, the occurrence of alternative binding modes of a CYP substrate can affect the catalytic activity at one substrate site if another binding mode, which is not productive for the particular reaction, is not specifically preferred. Accordingly, in addition to the observed substrate and enzymewise differences in the binding poses simulated here, the 7-hydroxylation activity of the 3-phenylcoumarins by the CYP1 enzymes can also be affected by alternative binding modes which the compounds may adopt.

The present MD simulations suggested a new binding mode for 3-phenylcoumarins compared with molecular docking carried out in this and previous work.^8^ Instead of the Ser122 or Thr124 H-bond with CYP1A1 and CYP1A2, the water-mediated H-bond from the 3-phenylcoumarin 2-carbonyl to the CYP1-conserved Asp313 (CYP1A1 and CYP1A2) or Asp326 (CYP1B1) was found (Figure 3). The Ser122/Thr124 H-bond is stably present in just five MD simulations of CYP1A1 in complex with compounds **13** and **15–23** (Table 3). The MD simulations of compounds **13–23** indicated that water channels open readily to the CYP1 binding sites, and the emerging waters can mediate crucial H-bonds between the ligand and the enzyme. Water-mediated H-bonds to the CYP1 enzymes also appears at the H-bonding groups of the 3-phenyl ring of the compounds. While the water-mediated interactions are crucial for the stabilization of the 3-phenylcoumarins to the CYP1 binding sites, water molecules can also destabilize the binding mode. Switching of water H-bonds and the mobile water network destabilizes, for example, **19** in complex with CYP1A1 and CYP1B1 (Figure 3A,C) and **20** in complex with CYP1A2 (Figure 3H). In summary, a limited number of water molecules can enter the binding sites of CYP1s, where they affect the binding of ligands.

CYP1A1 7-hydroxylated most of the 3-phenylcoumarin compounds **13–23** with high efficiency. CYP1A1 has very low 7-hydroxylation activity on only three of the compounds and has higher intrinsic clearance than CYP1A2 and CYP1B1 on three compounds (Table 1). Among the three CYP1 forms, CYP1A1 allows more water molecules at its binding site than CYP1A2 in the MD simulations in complex with compounds **13–23** (Table 2). Appropriately, the binding site volume of CYP1A1 is larger than that of CYP1A2 and CYP1B1.^8^ Bound with 3-phenylcoumarins, the large binding site allows more water molecules at the proximity of the compound. On the one hand, this can destabilize the binding mode, as demonstrated with **19** (Figure 3A). On the other hand, more 3-phenylcoumarins with varying substituents can find a suitable orientation for 7-hydroxylation in the larger binding site of CYP1A1. For example, **19** and **20** are both quite efficiently 7-hydroxylated, but they find a different angle and H-bonds with CYP1A1 (Figure 3A,G). Even the larger **14** can fit in the binding site (Figure 3D) and be 7-hydroxylated by CYP1A1 regardless of its shielding 6- and 8-chlorines, although with low activity (Table 1). Among CYP1 enzymes, CYP1A1 seems to be the most versatile oxidation catalyst of 3-phenylcoumarin compounds.

CYP1A2 was more efficient than CYP1A1 and CYP1B1 in the 7-hydroxylation of **15** and 7-hydroxylated all but one of the compounds **13–23**. In a complex with CYP1A2, the least number of water molecules flow to the proximity of the compounds **13–23** in the MD simulations among the three CYP1 forms (Table 2). This likely results in less destabilization of the binding poses by water molecules. The small number of water molecules at the binding site might result from the placement of the channels that open to the cavity during the MD simulations. In CYP1A1 and CYP1B1, the channel on the “left” side is most often located between Ser116/127 and Asn255/265 (CYP1A1/1B1) (Figure 3). In contrast, the channel is located closer to the heme between Thr118 and Asp313 in the CYP1A2 simulations. Another factor that can reduce the number of water molecules at the CYP1A2 binding site is that its cavity volume might be better suited for the tested 3-phenylcoumarins than the one of CYP1A1 and CYP1B1.^8^ Consequently, the compounds could fill the binding site of CYP1A2 perfectly, without poking into channels or allowing an excessive amount of water molecules in. Finally, a hydrophobic nook above the I chain, not found in CYP1A1 or CYP1B1,^8^ is likely a perfect compartment for hydrophobic substituents at the phenyl ring of 3-phenylcoumarins. As the access of water molecules to the CYP1A2 binding site is restricted, the placement of water molecules is more critical for 3-phenylcoumarin binding than in CYP1A1 or CYP1B1.

CYP1B1 was the least efficient at 7-hydroxylation of the tested 3-phenylcoumarins **13–23** (Table 1). The number of water molecules near the simulated compounds (Table 2) and the channels that open during the MD simulations (Figure 3) are very similar to CYP1A1. In addition, the shape of the CYP1B1 binding site is very similar to CYP1A1; however, CYP1B1 has the smallest binding site among the three CYP1 forms.^8^ Therefore, the occurrence of the 7-hydroxylation reaction is likely more sensitive to the exact shape of the compound. With the correct size, shape, and H-bonding groups, CYP1B1 can still be unmatched in the efficiency of 7-hydroxylation of certain 3-phenylcoumarins. In the MD simulation of **20** in complex with CYP1B1, the stable H-bond from the 4′-acetoxy shows that a correctly placed H-bonding group at the 3-phenyl ring can remarkably boost the efficiency of 7-hydroxylation by CYP1B1. Although CYP1B1 can also 7-hydroxylate some more hydrophobic 3-phenylcoumarins such as **19** with high efficiency (Table 1), the H-bond from **20** to the unique Gln332 advances the reaction. In summary, the most critical features of 3-phenylcoumarin binding to CYP1B1 seem to be its differing H-bonding amino acids and the smaller size of the binding site as compared to CYP1A1 and CYP1A2.

Water molecules may have an important role in ligand recognition and binding in CYP1 enzymes. Water molecules are found in all CYP1A1,^8^ CYP1A2,^9^ and CYP1B1^10^ crystal structures at the binding site or at its immediate proximity. In addition, a water-mediated H-bond is reported from α-naphthoflavone to CYP1A2.^9^ Inclusion of the CYP1A2 binding site crystal water has also been identified to improve the prediction of substrate binding modes and sites of metabolism in molecular docking, although the same water position is not optimal for all ligands.^51^ As in the present MD simulations, previous simulations of CYP1A2 have shown that water molecules emerge readily to the CYP1A2 binding site. In addition, water networks differ between CYP1A2 ligands.^52^ Here, water molecules emerged at the binding sites of all three CYP1 enzymes in the MD simulations, and they had close interactions with the 3-phenylcoumarin ligands.

Coumarin and its numerous derivatives are commonly used as profluorescent CYP substrates. These include 7-ethoxycoumarin, 3-cyano-7-ethoxycoumarin, 7-ethoxy-4-trifluoromethylcoumarin, 7-methoxy-4-trifluoromethylcoumarin, 7-methoxy-4-aminomethylcoumarin, and 7-benzyloxy-4-trifluoromethylcoumarin. The shortcoming of these substrates is that they are not selective but are oxidized by several CYP forms.^15,53^ Especially, 7-ethoxycoumarin O-deethylation is well known to be mediated by multiple human CYP forms. CYP1A1 catalyzes the reaction with the highest efficiency, followed by CYP1A2, CYP2E1, CYP2A6, and CYP2B6.^16^ 7-Ethoxyresorufin is the classical selective probe substrate of all CYP1 enzymes. It was oxidized more efficiently by CYP1A1 than by CYP1A2 or CYP1B1, as shown earlier.^54^ Reaction phenotyping in vitro with 7-ethoxyresorufin is the semiquantitative in vitro estimation of the relative contributions of CYP1-specific drug-metabolizing enzymes to the metabolism of a test compound.^55,56^

Fluorescence-based CYP assays are applied for two main purposes as follows: (1) measuring CYP-mediated activities in whole tissue samples or cellular fractions prepared from them or with recombinant or purified enzymes and (2) using the assay as a test compound independent method to screen for potential inhibition liability of CYPs by new drug candidates. Regarding the first application, fluorescence-based methods with coumarin substrates are sensitive, fast, reliable, simple, and low cost.^14^ Determination of CYP1 activity is integrated into modern toxicological concepts and testing guidelines, emphasizing the importance of this enzyme for risk assessment and regulation of chemicals.^57^ The second application arises from the need to detect CYP-mediated drug–drug interaction liability of drug candidates early in the drug discovery process. Numerous harmful interactions occur between drugs and other substances, and CYP inhibition is a major mechanism for such interactions. High-throughput fluorescence-based assays are today routinely carried out to screen the inhibitory potencies of a wide range of drugs and other substances.^14,53^

## 5 Conclusions

We developed 11 novel 3-phenylcoumarin derivatives for CYP substrates of which compound **19** was oxidized by very high efficiency by all three human CYP1 forms and displayed similar binding in the enzyme active sites. Compound **14** was selectively oxidized by CYP1A1, displaying better orientation and stronger H-bond interactions in the active site of CYP1A1 versus CYP1A2 and CYP1B1. Oxidation of **20** was catalyzed most efficiently by CYP1B1, explained in part by differences in stabilities of complexes between **20** and the three CYP enzymes. The sizes of binding sites, the key interactions, and the number and networks of water molecules explained differences of oxidation of 3-phenylcoumarins among three human CYP1 enzymes.

In this study, the catalytic properties of human CYP1A1, CYP1A2, and CYP1B1 enzymes were analyzed head-to-head by enzymological and modeling approaches. Compound **14** is a promising selective substrate for identifying CYP1A1 activity in tissues with low CYP3A4/5 content, and **20** is a novel high-efficiency substrate for measuring extrahepatic CYP1B1 activity. Compound **19** would be a good high-affinity CYP1 substrate in assays using recombinant human CYPs.

## Supporting Information Available

Details of enzyme kinetics including Michaelis–Menten graphs and details of binding energies and docking poses (PDF)
