# Supplementary material for: Substrate Selectivity of Coumarin Derivatives by Human CYP1 Enzymes: In Vitro Enzyme Kinetics and In Silico Modeling
Source: ACS Omega. 2021 Apr 19;6(17):11286–96. doi: 10.1021/acsomega.1c00123 (PMC8153946; doi:10.1021/acsomega.1c00123)
Supplement: Supplementary file 1 — ao1c00123_si_001.pdf [file ao1c00123_si_001.pdf]

Supporting information

Substrate selectivity of coumarin derivatives by human CYP1 enzymes: in vitro enzyme kinetics and in silico modelling

Risto O. Juvonen[1,*], Mira Ahinko[2], Elmeri M. Jokinen[3], Juhani Huuskonen[4], Hannu Raunio[1], Olli T. Pentikäinen[2,3,*]

[1]School of Pharmacy, Faculty of Health Sciences, University of Eastern Finland, Box 1627, 70211 Kuopio, Finland

[2]University of Jyvaskyla, Department of Biological and Environmental Science & Nanoscience Center, P.O. Box 35, FI-40014 University of Jyvaskyla, Finland

[3]Institute of Biomedicine, Faculty of Medicine, Integrative Physiology and Pharmacology, University of Turku, Kiinamyllynkatu 10, FI-20520 Turku, Finland

[4]University of Jyvaskyla, Department of Chemistry, P.O. Box 35, FI-40014 University of Jyvaskyla, Finland

*Correspondence should be addressed to Risto O. Juvonen (risto.juvonen@uef.fi)

Supplementary results

The coumarin compounds evaluated here are based on our previous experience of 3-phenylcoumarin derivatives being excellent profluorescent probes for several CYP forms.[1] Thus the 3-phenylcoumarin scaffold was promising for the design of selective profluorescent substrates for the CYP1 family enzymes. The three CYP1 forms can accommodate the 3-phenylcoumarin core in their binding sites so that coumarin position 7 lies close to the heme, leading to oxidation of the derivative to a fluorescent 7-hydroxylated product. Molecular modeling suggested that CYP1A1 and CYP1A2 stabilize the 3-phenylcoumarin core to favorable 7-hydroxylation coordinates by an H-bond from the coumarin carbonyl to Ser122 (CYP1A1) and Thr124 (CYP1A2). CYP1A1 and CYP1A2 were found to favor hydrophobic substituents such as methoxy and trifluoromethyl groups at the 3' and 4' positions of the 3-phenyl ring. In contrast, among the tested compounds, hydroxylation of the 3-phenylcoumarin position 7 by CYP1B1 was found to require an H-bonding group at the 3-phenyl ring, favorably at position 4'. Based on molecular docking, the 3-phenyl H-bonding group could bond with CYP1B1 Asn265. For 7-hydroxylation of 3-phenylcoumarin by CYP1A1 and CYP1A2 there needs to be a substitution at position 6 or 7 of the 3-phenylcoumarin scaffold [Juvonen et. al, 2019]. Figure S1 shows the Michaelis-Menten analysis of studied compounds.

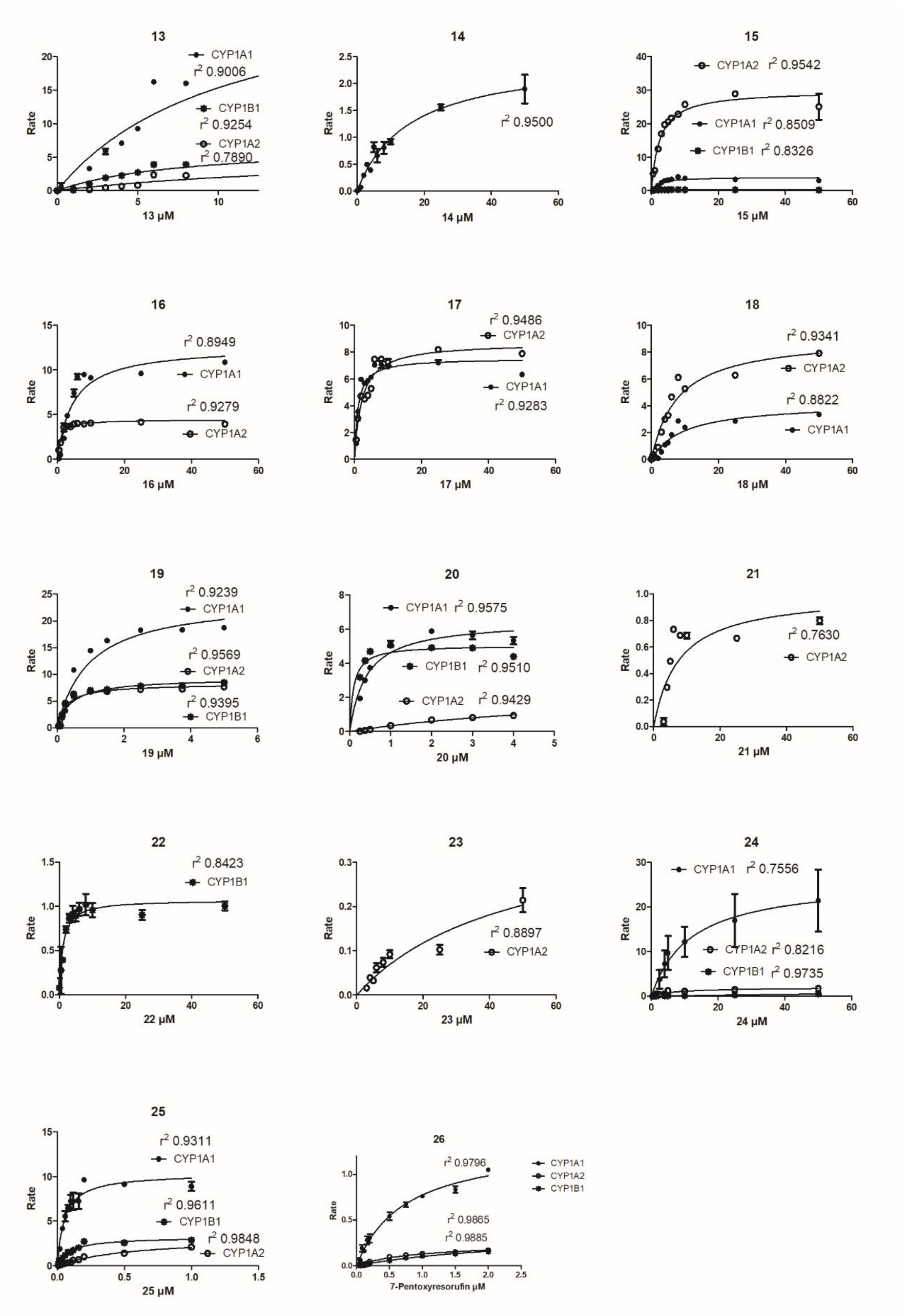

Figure S1. Michaelis-Menten graphs of oxidation of coumarin derivatives or ethoxy- or pentoxyresorufin by human CYP1A1, CYP1A2 and CYP1B1. Formation of fluorescent metabolites were determined in incubations containing 2–10 nM CYP, 20% NADPH regenerating system and 0.01–50 μM substrate in 100 mM Tris-HCl pH 7.4. The rate unit is mol product/(min*mol CYP).

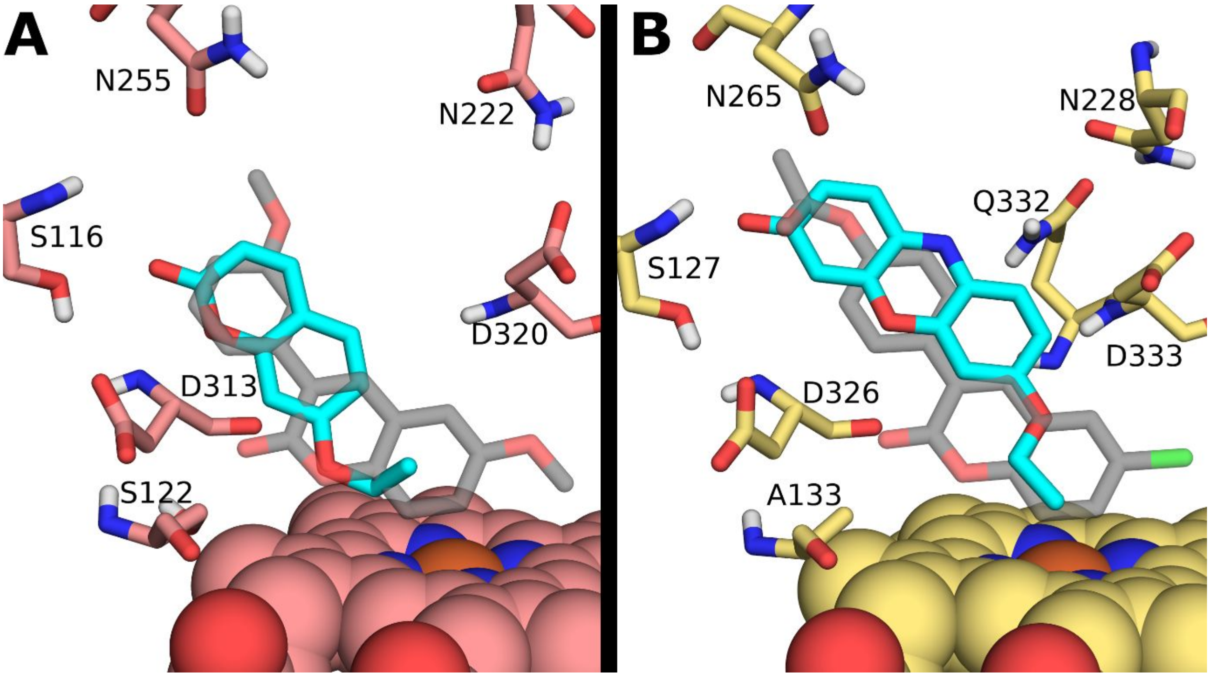

Figure S2. A. Molecular docking of 7-ethoxycoumarin (cyan carbon atoms) and compound **19** (grey transparent carbon atoms) to CYP1A1. B. Molecular docking of 7-ethoxyresorufin (cyan carbon atoms) and compound **20** (grey transparent carbon atoms) to CYP1B1.

Binding of compounds 13–23 to CYP1A1, CYP1A2 and CYP1B1

The binding interactions of **13–23** with the CYP1 enzymes were studied with molecular dynamics (MD) simulations, which were initiated from selected docking poses. The resulting binding free energy estimations (Table S1) were in favor of the binding poses. In the simulations, water molecules entered to the CYP1 binding sites (Table 2). The general orientation of the compounds stayed close to the starting poses (Table S2) and the position 7 remained close to the heme iron (Table 3). The interactions of **19**, **14** and **20** with the CYP1 enzymes are discussed in more detail below.

Table S1. The Nwat-MMGBSA igb5 binding energies with standard deviations of 3-phenylcoumarins in complex with the CYP1 forms.

Compound	N = 10 (kcal/mol)			N = 20 (kcal/mol)		
	CYP1A1	CYP1A2	CYP1B1	CYP1A1	CYP1A2	CYP1B1
**13**	-32.6±3.0	-37.7±2.6	-33.2±3.8	-32.4±3.0	-37.8±2.6	-33.7±3.8
**14**	-44.0±2.5	-38.2±2.9	-41.0±3.1	-44.4±2.5	-38.8±3.0	-41.8±3.0
**15**	-29.4±3.1	-29.9±2.5	-36.1±2.6	-28.7±3.2	-29.9±2.5	-35.8±2.6
**16**	-39.7±3.4	-41.0±3.0	-41.2±2.8	-39.6±3.5	-41.2±3.0	-41.8±2.8
**17**	-33.8±6.2	-39.2±2.5	-36.5±2.9	-33.8±6.4	-39.2±2.5	-37.2±2.9
**18**	-43.3±3.0	-38.4±3.7	-42.8±2.4	-43.4±3.0	-38.4±3.9	-43.1±2.4
**19**	-40.3±3.2	-41.0±2.5	-38.3±2.9	-39.9±3.2	-41.1±2.5	-38.5±2.9
**20**	-40.0±3.6	-42.9±2.6	-40.0±2.9	-40.4±3.7	-43.2±2.5	-40.9±2.9
**21**	-33.8±2.7	-36.5±2.1	-32.2±2.3	-33.8±2.8	-36.6±2.1	-32.7±2.3
**22**	-42.2±2.9	-38.5±2.5	-41.7±2.5	-42.5±2.9	-38.6±2.5	-42.2±2.5
**23**	-36.8±2.5	-33.0±2.8	-29.6±2.8	-36.7±2.5	-33.0±2.8	-29.8±2.8
**Average**	-37.8	-37.8	-37.5	-37.8	-38.0	-38.0

N: The count of closest water molecules to the 3-phenylcoumarin ligand as defined in the Nwat-MMGBSA method.[2-4]

Table S2. Combined root mean square deviation (RMSD) of the 3-phenylcoumarins and the CYP1 heme ($RMSD_{LH}$) with standard deviations.

Compound	$RMSD_{LH}$ (first frame)			$RMSD_{LH}$ (previous frame)		
	CYP1A1	CYP1A2	CYP1B1	CYP1A1	CYP1A2	CYP1B1
13	1.26±0.24	1.04±0.14	1.29±0.34	0.83±0.14	0.80±0.13	0.84±0.17
14	1.14±0.18	1.15±0.12	0.90±0.14	0.78±0.12	0.83±0.12	0.79±0.14
15	1.20±0.22	0.99±0.20	1.02±0.19	0.80±0.14	0.79±0.14	0.75±0.12
16	1.33±0.34	1.38±0.15	1.19±0.18	0.87±0.14	0.88±0.13	0.84±0.14
17	1.23±0.21	1.38±0.18	0.98±0.13	0.86±0.14	0.89±0.16	0.85±0.12
18	1.00±0.14	1.31±0.16	0.90±0.11	0.84±0.13	0.89±0.14	0.78±0.11
19	1.13±0.15	0.99±0.13	1.93±0.28	0.87±0.14	0.84±0.12	0.89±0.16
20	1.26±0.30	1.52±0.22	1.02±0.21	0.84±0.15	0.78±0.12	0.80±0.13
21	0.95±0.13	1.00±0.18	2.03±0.29	0.82±0.13	0.85±0.15	0.88±0.18
22	1.20±0.16	1.15±0.21	1.00±0.15	0.80±0.14	0.84±0.12	0.79±0.14
23	0.79±0.15	1.05±0.17	1.10±0.18	0.75±0.12	0.79±0.12	0.77±0.15
Average	1.14	1.18	1.21	0.82	0.84	0.82

Hydrophobic 19 is best stabilized by CYP1A2

Compound **19** is 7-hydroxylated by each three CYP1 form with high efficiency (Table 1). CYP1A2 catalyzes the 7-hydroxylation of **19** with the lowest K_m (0.27 µM) and the highest intrinsic clearance (V_{max}/K_m) value of 30.4 ML/(min * mol CYP) (Table 1). CYP1A1 comes next with the highest V_{max} and intrinsic clearance of 25.2. CYP1B1 catalyzes the 7-hydroxylation of **19** with the lowest intrinsic clearance. The methoxy substituents at positions 6 and 3' make **19** a rather hydrophobic and medium-sized among the tested 3-phenylcoumarin compounds **13**–**23** (Figure 1).

Inspection of the MD simulations of **19** are in line with the experimental results. **19** binds CYP1A2 in the most favorable way for 7-hydroxylation among the three CYP1 forms. The Nwat-MMGBSA is the best for **19** in complex with CYP1A2, although the values of CYP1A1 and CYP1B1 simulations are within error ranges (Table S1). The distance between **19** position 7 and the heme iron is lowest for the CYP1A2 complex (Table 3). In addition, the standard deviations of the measured distances are smallest in complex with CYP1A2, which indicates greater stability of **19** with CYP1A2 than with CYP1A1 and CYP1B1. In the MD simulations, **19** forms water-mediated H-bonds to each CYP1 and these H-bonds are the most invariable with CYP1A2.

In complex with CYP1A1, mobile water molecules in the binding site decrease the stability of **19**. Multiple waters mediate H-bonds from the **19** 2-carbonyl to Asp313, Ser116 and Asn255 (Figure 3A). These H-bonds draw **19** slightly away from the heme, position 7 being 5.0 ± 0.5 Å from the heme iron (Table 3). The water-mediated H-bonds are not well stabilized due to the mobility of the water molecules, which decreases stability of the whole **19** in the CYP1A1 binding site. These waters emerge from a channel between Ser116 and Asn255 (Figure 3A). In addition, multiple water molecules flow close to **19** from a channel between Asn222 and Asp320, which likely adds to the destabilization of the compound. The average number of water molecules within 3.4 Å of **19** is 7.0 ± 1.8 which is higher than for CYP1A2 and CYP1B1 (Table 2).

In complex with CYP1A2, **19** forms a single stable water-mediated H-bond from 2-carbonyl to Asp313 (10/10 frames) and Thr118 (8/10 frames) (Figure 3B). The H-bond is very stable as **19** fills the CYP1A2 binding site so that only a single water molecule is at the immediate proximity of the compound on that side of the binding site. This is possible because the water channel originates from between Thr118 and Asp313, which is different from CYP1A1. Other

water molecules from a channel between Thr223 and Asp320 do not interfere with the binding mode. Accordingly, the number of water molecules at the proximity of **19** is only 1.9 ± 0.6 in the CYP1A2 binding site (Table 3). Molecule **19** is snugly packed inside the otherwise hydrophobic binding site.

The interactions of **19** in the CYP1B1 binding site resemble those in CYP1A1. The water channel on the 2-carbonyl side of **19** lies between Ser127 and Asn265 (Figure 3 C), similar to the CYP1A1 simulation (Figure 3A). The 2-carbonyl forms alternating water-mediated H-bonds to Asp326, Ser127 and Asn265. These H-bonds draw **19** far from the heme (Table 3) and make the exact orientation of **19** waver. Adding to the effect, water molecules from a channel between Asp333 flow close to **19**. Although the average number of water molecules (4.1 ± 1.5) next to **19** is lower than in the CYP1A1 complex (Table 2), those water molecules flow even between the heme and **19** towards the end of the simulation.

A comparison of **19** to its structural kin **16** reveals similarities in the binding mode preference and 7-hydroxylation activity by the three CYP1 forms. The compounds share the 6-methoxy group, but instead of another methoxy group at position 3’, **16** has a large trifluoromethoxy group at position 4’. Similar to **19** although with lower efficiency, **16** is most readily 7-hydroxylated by CYP1A2, followed by CYP1A1 (Table 1). 7-hydroxylation by CYP1B1 occurs only with very low activity (Table 1). Accordingly, **16** is best stabilized by CYP1A2, as suggested by the small standard deviations of the distances of **16** positions 7 and 2 to the CYP1 forms (Table 3). The detailed interactions of **16** with the CYP1 forms are similar to **19**. The **16** 4’-trifluoromethyl finds a hydrophobic nook next to Thr223 at the CYP1A2 binding site, and the compound is neatly packed in the cavity. The water-mediated H-bond to CYP1A2 Asp313 is not quite as stable as for **19**, and the position 7 is 0.6 Å farther away from the heme iron on average (Table 3). In CYP1A1, the **16** 2-carbonyl shuffles water-mediated H-bonds similarly to **19**. In addition, the water-filled channel between Ser116 and Asn255 (Figure 3A) occasionally continues between Ser116 and Asp313. In complex with CYP1B1, the environment for the 4’-trifluoromethyl is the least suitable. First, the subcavity next to Asn228 is the most hydrophilic among the CYP1 forms. Second, the large size of the 4’ group encourages a channel to open on that side of the CYP1B1 binding site, which leaves the hydrophobic group exposed to waters outside the binding site. This leads to weak stabilizing interactions of **16** with CYP1B1 in this particular binding mode that would allow 7-hydroxylation.

14 is the most stable and accessible for oxidation at the CYP1A1 binding site

In terms of 7-hydroxylation, the most significant deviation of **14** from the other 3-phenylcoumarins is two protecting chlorines on both sides of position 7 at positions 6 and 8 (Figure 1). Accordingly, the coumarin core needs to be in exactly the correct angle in relation to the heme for position 7 to be available for hydroxylation. At the phenyl ring, **14** has a 3'-fluorine substituent and an acetoxy group at position 4'. **14** is 7-hydroxylated selectively by CYP1A1 and with only diminishing activity by CYP1B1 (Table 1).

In the MD simulations of **14** in complex with the CYP1 enzymes, the most favorable interactions for 7-hydroxylation of **14** are found in complex with CYP1A1, which is in line with the experimental results. By visual inspection, the binding interactions of **14** are equally good in CYP1A1 and CYP1B1. However, with CYP1A1 the binding energy (Table S1) and stability of **14** are better and the position 7 is more accessible to the heme iron (Table 2). The amount of ligand-water H-bonds and waters near **14** are similar in CYP1A1 and CYP1B1 (Table 1). In complex with CYP1A2, position 7 of **14** is the farthest from the heme iron (Table 2) and the binding energy (Table S1) and interactions are not as good as with CYP1A1 and CYP1B1.

The simulated binding pose of **14** is highly stable with CYP1A1. The 2-carbonyl forms H-bond via water to Asp320 (8/10 frames) and Thr497 (4/10 frames) (Figure 3D). The water-occupied site next to Asp320 and Thr497 is highly stable. The 4'-acetoxy group forms H-bonds via one water molecule to Asn255 (5/10 frames; Figure 3D) or via two water molecules to Asp313 (3/10 frames). Position 7 is well exposed to the heme iron due to the upright orientation of **14** in the binding site.

In complex with CYP1A2, **14** does not form any H-bonds and the 6-chlorine lies between position 7 and the heme iron (Figure 3E). Few water molecules enter the binding site (Table 2) and those few do not occupy the site next to the **14** 2-carbonyl, Asp320 and Thr498.

The orientation of **14** in complex with CYP1B1 is similar with the CYP1A1 complex. The 2-carbonyl forms H-bond via water to Asp333 (8/10 frames) and Thr510 (7/10 frames) (Figure 3F). Like in CYP1B1, position 7 of **14** is well accessible to the heme iron. The 4'-acetoxy group forms H-bond straight (9/10 frames) or via water (1/10) frames to Gln332.

20 finds the best H-bonds with CYP1B1

The array of the tested 3-phenylcoumarins to be more efficiently 7-hydroxylated by CYP1B1 than by 1A1 and 1A2 is scarce. 20 outstands with very high 7-hydroxylation efficiency by

CYP1B1 with an intrinsic clearance of 53 (Table 1). For CYP1A1 and 1A2 the intrinsic clearance is 16 and 0.6, respectively. For each CYP1 form the V_{max} is relatively low with the highest value 6.5 for CYP1A1. The 20 has 6-chlorine group and a 4'-acetoxy that can act as an H-bond acceptor at the 3-phenyl ring (Figure 1).

The simulated **20** binding mode is best stabilized by CYP1B1, although other numerical metrics do not show solid preference for **20** by CYP1B1. Position 7 is clearly closest to the heme iron in complex with CYP1A1, but positions 2 and 7 are best stabilized by CYP1B1 (Table 3). The Nwat-MMGBSA is lowest in the CYP1A2 complex (Table S1). The amount of water molecules at the proximity of **20** is similar among the CYP1 forms, but in complex with CYP1B1, **20** forms more hydrogen bonds with water molecules on average (Table 2).

The visual analysis reveals that in complex with CYP1A1, the **20** 2-carbonyl forms a fairly stable H-bond with CYP1A1, but the 4'-acetoxy shuffles between different H-bond donors and causes fluctuation in the binding mode. After an initial H-bond to a water molecule from the channel between Ser116 and Asn255 (3/10 frames), the 2-carbonyl H-bonds with Ser122 (7/10 frames; Figure 3 G). The Ser122 H-bond draws the 6-chlorine in **20** close to the hydrophobic periphery of the CYP1A1 binding site. The 4'-acetoxy forms H-bond to Asn255 (4/10 frames) or different water molecules in two main water channels that open to the CYP1A1 binding site (6/10 frames; Figure 3G). The shuffling H-bonds of the 4'-acetoxy likely make the **20** more unstable with CYP1A1 than it is with CYP1B1.

Compound **20** is the least stabilized in complex with CYP1A2. The 4'-acetoxy forms an H-bond with Asn257 (7/10 frames; Figure 3 H). Combined with the shape of the CYP1A2 binding site, the Asn257 H-bond tilts **20** in a more upright position in the CYP1A2 binding site as compared to CYP1A1 (Figure 3G) and CYP1B1 (Figure 3I). Consequently, an H-bond to Thr124 (1/10 frames) does not endure and, instead, the 2-carbonyl forms a water-mediated H-bond to Asp313 by one (7/10 frames; Figure 3H) or more water molecules (2/10 frames). In addition, the mobile water molecule network from the Asp313 channel destabilizes the binding mode. The instability is also demonstrated by the large standard deviation of the distance from the 2-carbonyl to Thr124 (Table 3).

Compound **20** forms the most stable interactions with CYP1B1. In the MD simulation, the **20** 2-carbonyl forms a stable water-mediated H-bond to Asp326 (8/10 frames; Figure 3I). The 4'-acetoxy H-bonds straight (5/10) or via a water molecule (3/10) to Gln332, which is unique for CYP1B1. With these H-bonds from the 2-carbonyl and the 4'-acetoxy, **20** is drawn into a

position where the 6-chlorine is neatly packed against the hydrophobic periphery of the CYP1B1 pocket. The water-occupied sites are quite firmly placed with no excess water molecules coming to the binding site.

A comparison of the interactions of **20** and its closest structural relative **22** reveals something about the details of CYP1B1 preference of an H-bonding group at position 4' over position 3'. **22** differs from **20** only by the acetoxy being at position 3' instead of 4' (Figure 1), yet it 7-hydroxylated with much lower efficiency by CYP1B1 than **20** (Table 1). In the MD simulation in complex with CYP1B1, **22** lacks the amount and stability of H-bonds to the enzyme that the **20** holds. The 3'-acetoxy of **22** occupies the same area of the CYP1B1 binding site (not shown) as **20** (Figure 3I). There, **22** forms a water-mediated H-bond to Asn265 (6/10 frames) instead of Gln332. Regardless of the exact H-bonding partner, the location of the 3'-acetoxy tilts the **22** so that the 3-phenylcoumarin core blocks the way of any waters from the channel between Ser127 and Asn265 that could mediate an H-bond from the 2-carbonyl to Asp326, which is seems essential for **20** (Figure 3 I). Respectively, the 2-carbonyl of **22** lies 2.0 Å closer to Ala133 than the 2-carbonyl of **20**, while the position 7 keeps the same distance to the heme iron for both compounds (Table 3). In short, the small difference in the position of the H-bonding group at the 3-phenyl ring leads to significant shift in the orientation of the compound in the CYP1B1 binding site, which prevents crucial water molecules to fit into the binding site and mediate an H-bond from the 2-carbonyl. However, **22** stays well in place in the CYP1B1 binding site, as shown by the small standard deviation of the position 2 and 7 distances (Table 3), and the 7-hydroxylation reaction is possible with low efficiency (Table 1).

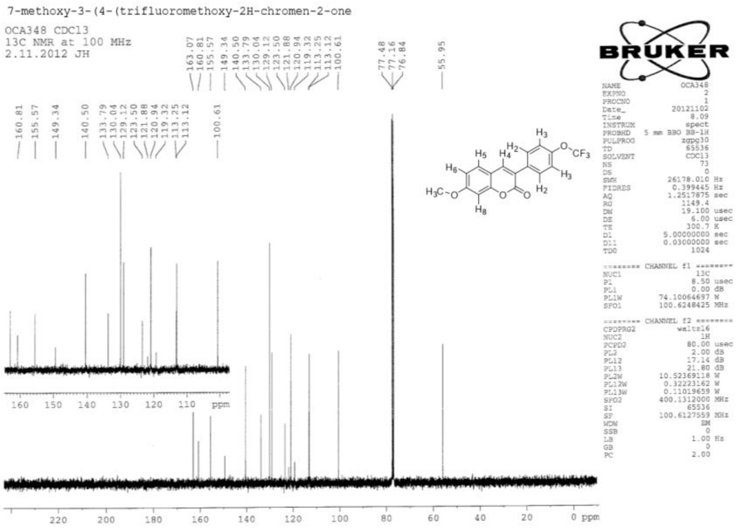
7-methoxy-3-(4-(trifluoromethoxy-2H-chromen-2-one
OCA348 CDCl3
13C NMR at 100 MHz
2.11.2012 JH
163.07
160.81
155.57
149.34
140.50
133.79
130.04
129.12
123.50
121.88
120.94
119.32
113.25
113.12
100.61
77.48
77.16
76.84
55.95
BRUKER
ppm

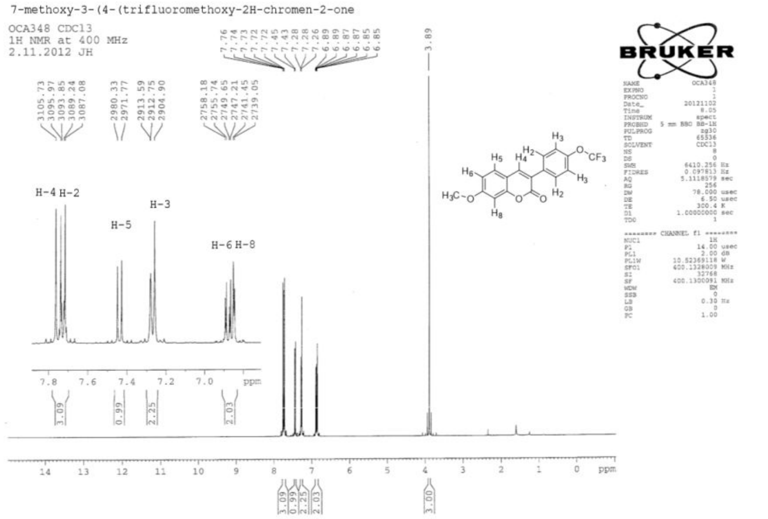
7-methoxy-3-(4-(trifluoromethoxy-2H-chromen-2-one
OCA348 CDCl3
1H NMR at 400 MHz
2.11.2012 JH
3.89
H-4 H-2
H-5
H-3
H-6 H-8
BRUKER
ppm

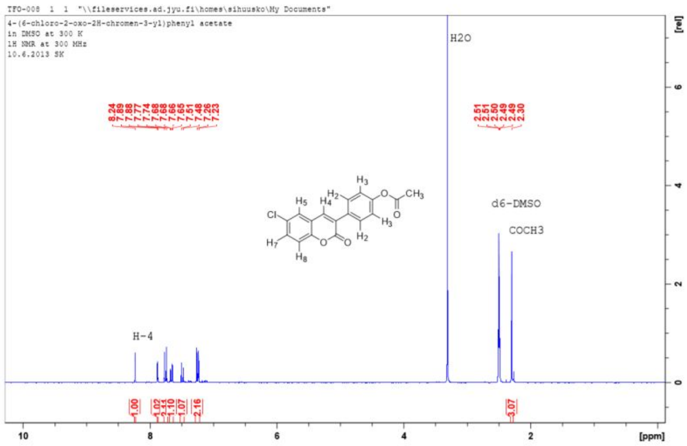
4-(6-chloro-2-oxo-2H-chromen-3-yl)phenyl acetate
in DMSO at 300 K
1H NMR at 300 MHz
10.6.2013 SK
H2O
d6-DMSO
COCH3
H-4
[ppm]
[rel]

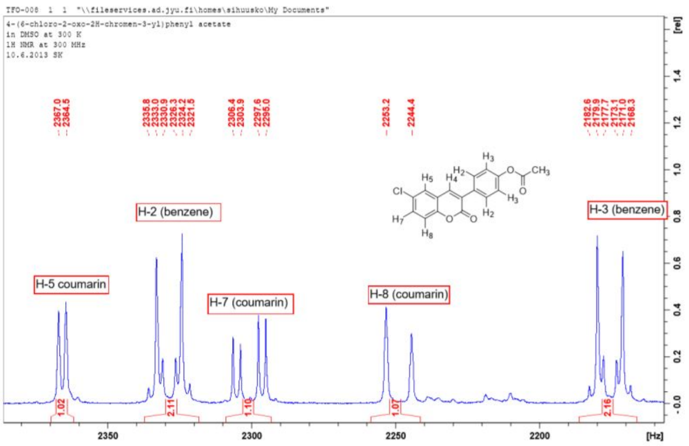

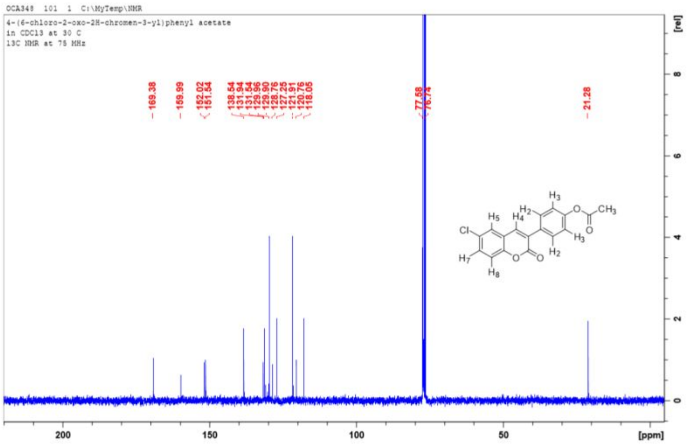

Figure S3. NMR spectra of compounds **18** **(**3-(4-trifluoromethoxyphenyl)-7-methoxycoumarin) and **20** (3-(4-acetoxyphenyl)-6-chlorocoumarin).

REFERENCES

(1) Juvonen, R. O.; Ahinko, M.; Huuskonen, J.; Raunio, H.; Pentikäinen, O. T. Development of new coumarin-based profluorescent substrates for human cytochrome P450 enzymes. *Xenobiotica* **2019**, *49*, 1015–1024.

(2) Maffucci, I.; Contini, A. Explicit Ligand Hydration Shells Improve the Correlation between MM-PB/GBSA Binding Energies and Experimental Activities. *J. Chem. Theory Comput.* **2013**, *9*, 2706−2717.

(3) Maffucci, I.; Contini, A. Improved computation of protein–protein relative binding energies with the Nwat-MMGBSA method. *J. Chem. Inf. Model.* **2016**, *56*, 1692–1704.

(4) Maffucci I.; Hu X.; Fumagalli V.; Contini A. An Efficient Implementation of the Nwat-MMGBSA Method to Rescore Docking Results in Medium-Throughput Virtual Screenings. *Front. Chem.* **2018**, *6*, 43.
